# Chlamydia pneumoniae infection and cerebrovascular disease: a systematic review and meta-analysis

**DOI:** 10.1186/1471-2377-13-183

**Published:** 2013-11-21

**Authors:** Juan Chen, Meijia Zhu, Gaoting Ma, Zhangning Zhao, Zhongwen Sun

**Affiliations:** 1 Department of Nephrology, Affiliated Qianfoshan Hospital of Shandong University, 66 Jingshi Road, Jinan, Shandong province 250014, China; 2 Department of Neurology, Affiliated Qianfoshan Hospital of Shandong University, 66 Jingshi Road, Jinan, Shandong province 250014, China; 3 Department of Neurology, Yantai Yuhuangding Hospital, 20 Yudong Road, Yantai 264000, Shandong province, China

**Keywords:** Chlamydia pneumoniae, Infection, Cerebrovascular disease, Atherosclerosis, Meta-analysis

## Abstract

**Background:**

A wealth of published studies have been published on association between Chlamydia pneumoniae (C.pneumoniae) infection and cerebrovascular (CV) disease, but the results were inconsistent. This meta-analysis provides a systematic review of the available evidence from all serological and pathological studies of CV disease and C.pneumoniae.

**Methods:**

A comprehensive research was conducted of MEDLINE, EMBASE, CNKI, WanFang technological periodical database and reference lists of articles to identify eligible case-control and cohort studies. Odds radio (OR) was calculated for each study outcome. Random effect model was used as pooling method and publication bias was estimated for the results.

**Results:**

Fifty-two published studies that met criteria were selected. In case control studies, an association between C.pneumoniae infection and CV disease was revealed by serum specific IgG (OR, 1.61; 95% CI: 1.34 to 1.94), serum IgA (OR, 2.33; 95% CI: 1.76 to 3.08) and PCR technique of C.pneumoniae in peripheral blood cells (OR, 1.90; 95% CI: 1.17 to 3.07). No significant association was found in serum anti-C.pneumonae IgM seropositivity or in-situ-detection of C.pneumoniae in arterial biopsies with CV disease. Subgroup analysis by available studies suggested that C.pneumoniae may paly a role in atherosclerotic stroke, but be less significant in stroke of cardioembolism or other etiologies.

**Conclusion:**

Association between C.pneumoniae infection and CV disease depends on the analytical method adopted, which seems stronger with stroke due to large artery atherosclerosis. Establishing a causal relationship between C.peumoniae infection and CV disease will require more prospective studies with combination of techniques and stratified by etiological subtypes.

## Background

Cerebrovascular (CV) disease is one of the major causes of long-term disability and mortality throughout the world. Atherosclerosis is the underlying pathology responsible for CV disease in developed countries and remains a serious problem in developing nations [1]. Conventional risk factors (eg, hypertension, diabetes, dyslipidemia and smoking) can not completely explain the pathogenesis of this disease and many patients, especially younger patients usually lack these risk factors. Over the past decades, increasing body of evidences demonstrated that chronic viral and bacterial infection contributes to the development of atherosclerotic lesions [2]. C.pneumoniae is one of the mostly implicated pathogens in this process [3-5].

C.pneumoniae, an obligate intracellular gram negative bacterium, disseminates via respiratory secretion, causing about 10% of community-acquired pneumonia cases and 5% of bronchitis cases [6]. A study published in 1988 firstly proposed that C.pneumoniae infection was an avoidable cause of coronary heart disease [7]. Subsequently, considerable epidemiological studies implicated C.pneumoniae in atherogenic process of CV events, based on the evidence from the participation of this pathogen in anti-phospholipids antibody formation, oxidation of LDL, and proliferation of smooth muscle cells [4,5]. Clinical trials on the clinical burden of cardiovascular disease under the influence of antibiotic treatment have also been conducted. However, these observations triggered the subsequent publication of several other reports with conflicting results. More recently, a prospective cohort study have linked the combined activity of several infections (i.e. an infection burden), rather than single infection to stroke risk [8]. Therefore, despite the publication of numerous articles on the association, it remains controversial whether C.pneumoniae is an active player or “innocent bystander” for CV disease. Different types of study design and various laboratory tests may largely contribute to the disparate findings. Furthermore, the etiology of CV disease forms is distinct, it is necessary to investigate evidence of C.pneumoniae infection stratified by different stroke etiologies.

To fill the gap, we performed for the first time this meta-analysis of all eligible studies published before September 2012 to clarify if there is an association between chronic C.pneumoniae infection and CV disease risk; 2) investigate whether the association varies depending on different subtypes of CV disease; 3) evaluate whether the association depend on different materials or laboratory tests.

## Methods

### Literature search

We searched the MEDLINE, EMBASE, CNKI (China National Knowledge Infrastructure) and Wanfang technological periodical database for relevant studies using the following main MeSH heading: chlamydia pneumoniae, chlamydophyla pneumoniae, atherosclerotic, atherosclerosis, stroke, cerebral ischemic, cerebrovascular, cerebral accident, cerebral apoplexy. An upper date limit was September 2012 and the languages were restricted to Chinese and English. Additional references were identified by reviewing the bibliographies of retrieved articles. After an initial screening of titles and abstracts, only relevant articles remained. The full text of these publications was read to decide whether needed information on the topic of interest was included.

### Inclusion criteria

Articles were eligible if they met the following criteria: 1) the study evaluated the relation between C.pneumoniae infection and CV disease (defined as any fatal or non-fatal ischemic stroke,hemorrhagic stroke or transient ischemic attack); 2) the study had a (nested) case-control or cohort design; 3)validated testing tool(s) for detecting C.pneumoniae was used; 4) they reported risk estimate with 95% confidence interval (CI) or provided sufficient information to calculate. When more than one of the same patient population was included in several publications, only the most recent or complete study was included. No quality scoring was attempted, since the scoring in meta-analyses of observational studies is controversial and has not been internationally accepted to date [9]. Two investigators (Juan Chen and Meijia Zhu) independently selected studies and discrepancies were resolved by consensus.

### Data extraction

For eligible study, the following information was extracted independently by two researchers: first author’s name, publication year and country, study design type, average patient characteristics (age and male proportion of cases) within each study, assay methods of C.pneumoniae infection, fully adjusted risk estimate with the corresponding 95% CI and the adjustment for the main confounding factors. For studies in which risk estimates were reported for more than one set of adjustments, the most adjusted estimate was used. For the studies in which only the number of participants was presented, the unadjusted risk estimates and 95% CIs were calculated. For the sero-epidemiological studies using microimmunofluorescence (MIF) as test method, the criterion for the antibody cut-off point titer was 1/16 or higher.

### Statistical analysis

We present this meta-analysis following the Meta-analysis of Observational Studies in Epidemiology (MOOSE) statement [9]. The risk estimate as well as its lower and upper CIs were log transformed for odds ratios before conducting the analyses. The pooled value for the effect size (odds radio) was calculated by use of a random effect model using the method of DerSimonian & Laird [10]. Since heterogeneity could be present as the result of the variety of studies included. Heterogeneity was assessed with the use of the Q statistic, inconsistency index (I^2^), taking values in the range 0 ~ 100%, whereas p-value < 0.05 or I^2^ > 50% were considered as significant heterogeneity. We performed a subgroup analysis to assess the ORs in patients with stroke of different subtypes according to Trial of Org 10172 in Acute Stroke Treatment (TOAST) criteria. Meta-regression with restricted maximum likelihood (REML) was performed to explore the potentially important covariates: age (mean age in participants) and sex (proportion of males in cases), with the dependent variable being the natural logarithm of the OR from each study. Publication bias was evaluated using funnel plot, Egger’s regression and Begger’s method. In the presence of publication bias, the non-parametric “trim and fill” method of Duval and Tweedie [11] was applied as a type of sensitivity analysis in order to assess the potential influence of missing studies (if any). All statistical analyses were performed using STATA version 11.0 (Stata Corp, College Station, TX). Statistical significance was defined at p value < 0.05.

## Results

### Identified studies

**Figure 1:**
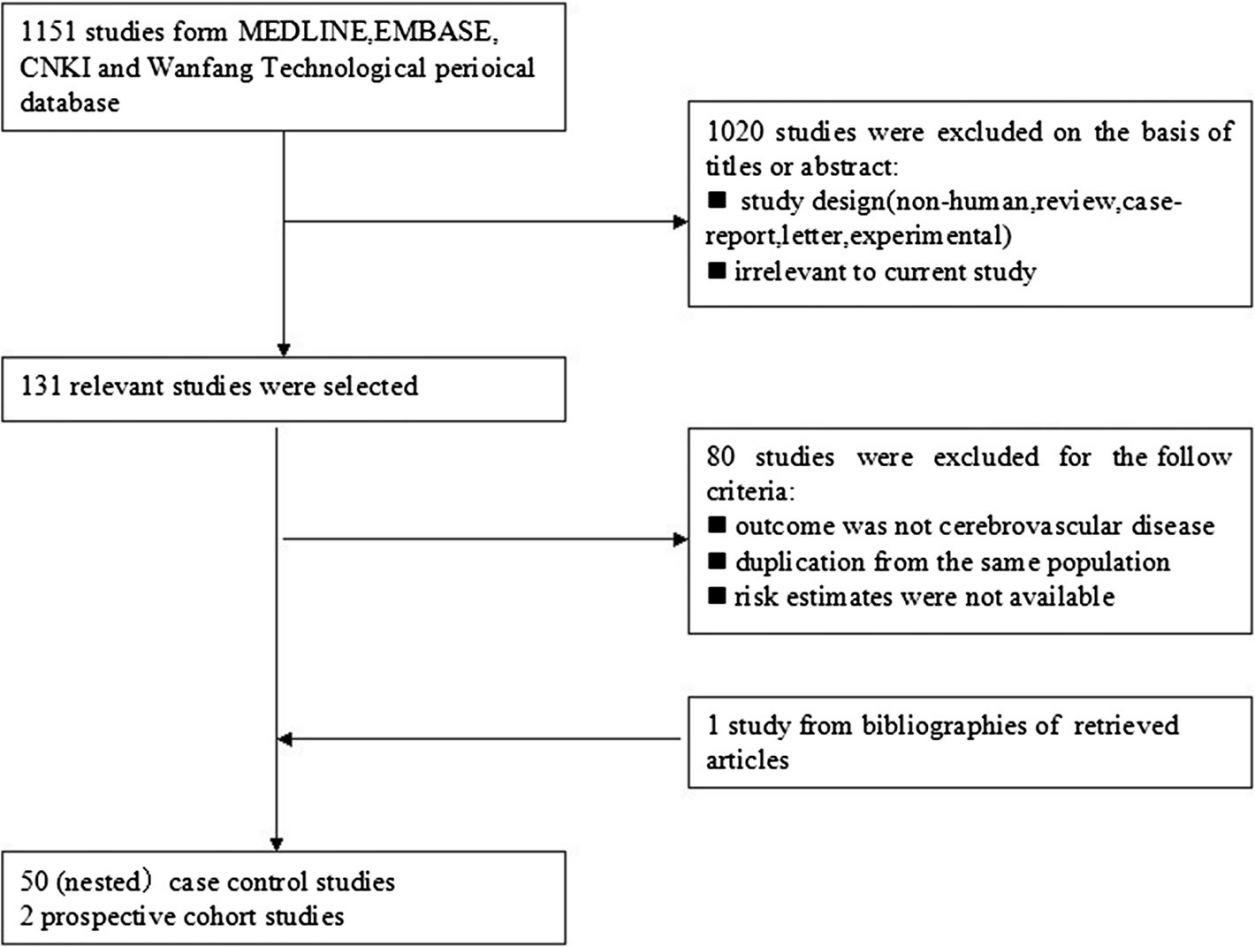
Flow diagram of the study-selection process.

Our search initially identified 1151 unique citations, of which the majority were excluded after the first screening of titles and abstracts, and mainly because they were reviews, case-reports, letters or irrelevant to our analysis. After assessing full text of potentially relevant articles, 52 studies published between 1996 and 2012 were enrolled [3-5,8,12-59], comprising 50 (nested) case-control studies and 2 prospective cohort studies. Figure 1 shows a flow diagram describing the study selection process. The majority of studies matched regarding various population characteristics and maximally adjusted estimates from studies were used instead of the raw data or unadjusted ones in some of included studies. The classification of the material was based on the allowable methods of measurement: polymerase chain reaction (PCR) technique, serum antibodies or immunohistochemical method (IHC) against C.pneumoniae. Most of the studies detected serum antibody to define C.pneumoniae infection and report summary statistics, as 12 studies used PCR in peripheral blood cells or IHC in carotid biopsies for validating C.pneumoniae detection. Table 1 and 2 demonstrate the main characteristics of these studies.

**Table 1:** Characteristic of serological studies

**Author Y**	**Country**	**Study design**	**Number of case/control**	**Case assessment or endpoint**	**Serologic testing assay**	**Laboratory test/Marker**	**Adjustment**
Bandaru VC 2012	India	CSCC	100/100	Ischemic stroke	MIF	IgA, IgG	Age, gender, diabetes, smoking, hypercholesterolemia, hyperhomocysteinemia.
Hasan ZN 2011	Iraq	CSCC	50/40	Ischemic stroke	ELISA	IgA, IgG	None
Kēnina V 2011	Latvija	CSCC	102/48	Ischemic stroke	ELISA	IgG	None
Chen YJ 2010	China	CSCC	50/50	Ischemic stroke	MIF	IgG	None
Bandaru VC 2009	India	CSCC	120/120	Ischemic stroke	MIF	IgA, IgG	None
Bandaru VC 2009	India	CSCC	120/120	Ischemic stroke	MIF	IgA, IgG	None
Tiszlavicz Z 2009	Hungary	CSCC	280/150	Ischemic stroke	ELISA	IgG	None
Alavi SM 2009	Iran	CSCC	45/45	Ischemic stroke	ELISA	IgG	None
Bandaru VC 2008	India	CSCC	200/200	Ischemic stroke	MIF	IgA, IgG	Age, diabetes, smoking, alcoholics, hypertension, hyperhomocystenemia, hypercholestemia.
Lin TM 2008	China	CSCC	450/450	Ischemic stroke	MIF	IgG	Gender, hypertension, diabetes, hypercholesterolemia, and smoking.
Hu J 2008	China	CSCC	100/60	Ischemic stroke, TIA	ELISA	IgG	None
Kis Z 2007	Hungary	CSCC	59/52	Ischemic stroke	ELISA	IgA, IgG	Age, gender
Alamowitch S 2007	France	CSCC	483/483	Ischemic stroke	MIF	IgA, IgG	Diabetes, smoking, hypertension, total cholesterol, CHD, season of enrolment.
Piechowski JB 2007	Poland	CSCC	94/103	Ischemic stroke	ELISA	IgA, IgG	Age and gender
Chi LQ 2007	China	CSCC	50/48	Ischemic stroke	ELISA	IgG	None
Liu JX 2006	China	CSCC	47/48	Ischemic stroke	ELISA	IgG	None
Njamnshi AK 2006	Cameroon	CSCC	64/64	Ischemic stroke, TIA	MIF	IgA, IgG	Diabetes, smoking, hypertension, obesity and alcohol intake.
Elkind MS 2006	USA	CSCC	239/428	Ischemic stroke	MIF	IgA, IgG	Age, gender, diabetes, smoking, atrial fibrillation, hypertension, HDL, LDL and race.
Chen L 2006	China	CSCC	135/135	Ischemic stroke	ELISA	IgG	None
Liu AM 2006	China	CSCC	80/80	Stroke	MIF	IgA, IgG	None
Zhou B 2005	China	CSCC	76/80	Ischemic stroke	EIA	IgA	Age, gender, smoking, alcohol intake, body weight, hypertension, cholesterol, hypertention.
Johsen SP 2005	Denmark	NCC	254/254	Ischemic stroke	ELISA	IgA, IgG	Diabetes, smoking, hypertension, cholesterol, BMI, alcohol intake, and education.
Li FL 2004	China	CSCC	65/25	Stroke	ELISA	IgA, IgG, IgM	None
Anzini A 2004	Italy	CSCC	141/192	Ischemic stroke	MIF	IgA, IgG, IgM	Age, gender, CV risk factors and socioeconomic status.
Manuela V 2004	Netherlands	CSCC	41/55	Ischemic stroke	ELISA	IgA, IgG	Smoking, hypertension and hypercholesterolemia.
Apfalter P 2004	Australia	CSCC	45/30	Stroke, TIA	MIF	IgA, IgG, IgM	None
Carusone CS 2004	Canada	CSCC	28/24	Stroke	MIF	IgA, IgG	Age, gender and smoking.
Bucurescu G 2003	USA	CSCC	25/25	Stroke, TIA	MIF	IgA, IgG	None
Ngeh J 2003	UK	CSCC	100/87	Stroke, TIA	ELISA	IgA, IgG, IgM	Age, gender, smoking, diabetes, hypertension, CHD.
Kawamoto R 2003	Japan	CSCC	40/85	Ischemic stroke	ELISA	IgG	None
Sirmatel F 2003	Turkey	CSCC	26/53	Ischemic stroke	MIF	IgG	None
Zou R 2003	China	CSCC	112/50	Stroke	ELISA	IgM, IgG	None
Wolf SC 2003	Germany	CSCC	30/116	Ischemic stroke	ELISA	IgA, IgG	Age, gender, diabetes, match for other risks factors.
Sessa R 2003	Italy	CSCC	18/33	Ischemic stroke,TIA	MIF	IgA, IgG	None
Smiega M 2003	Canada	NCC	107/3061	Stroke	MIF	IgA ,IgG	Age, gender, diabetes, smoking, hypertension, and hypercholesterolemia.
Tanne D 2003	Israel	NCC	134/134	Ischemic stroke	ELISA	IgA, IgG	Diabetes, smoking, BMI, hypertension.
Katie A C 2003	Australia	PC	119/1493	Stroke	MIF	IgA, IgG	Age, gender, diabetes, smoking, BMI, hypertension, cholesterol, TG, hemoglobin and systolic blood pressure.
Gerdes v 2002	Netherlands	NCC	26/273	Ischemic stroke	ELISA	IgA, IgG	None
Madre JG 2002	Spain	CSCC	91/112	Ischemic stroke	MIF	IgA	None
Tarnacka B 2002	Poland	CSCC	20/15	Sympotomatic carotid disease	ELISA	IgG	None
LaBiche R 2001	USA	CSCC	36/55	Sympotomatic carotid disease	MIF	IgA, IgG, IgM	None
Katsenis C 2001	Greece	CSCC	20/15	Sympotomatic carotid disease	ELISA	IgM	None
Elkind MS 2000	USA	CSCC	89/89	Ischemic stroke	MIF	IgA, IgG	Diabetes, smoking, hypertension, HDL, education, match for age, gender, race.
Glader CA 1999	Sweden	NCC	97/197	Ischemic stroke	MIF	IgA, IgG	Diabetes, smoking, BMI, hypertension, hypercholesterolmia.
Wimmer MLJ 1996	Germany	CSCC	58/52	Ischemic stroke,TIA	MIF	IgA, IgG	Age, gender, hypertension, migraine.

CSCC, cross-sectional case control; NCC, nested case control; PC, prospective case–control or cohort; ELISA, enzyme-linked immunosorbent assay; MIF, microimmunonuorescence; HDL ,high density lipoprotein; LDL, low density lipoprotein;TG, triglycerides; CHD, coronary heart disease; BMI, body mass index; TIA, transient ischemic attack; CV, cerebrovascular.

**Table 2:** Characteristic of studies using PCR or IHC

**Author Y**	**Country**	**Study design**	**Number of case/control**	**Case assessment**	**Detecting assay**	**Specimen**	**Odds ratio (95% CI)**
Gagliardi RJ 2009	Brazil	CSCC	52/59	Ischemic stroke, TIA	PCR	PBMC/DNA	0.37(0.01 ~ 9.32)
Kis Z 2007	Hungary	CSCC	59/52	Ischemic stroke	PCR	PBMC/DNA	1.92(0.61 ~ 6.01)
Apfalter P 2004	Australia	CSCC	45/30	Stroke, TIA	PCR	PBMC/DNA	0.65(0.09 ~ 4.89)
Wang SL 2003	China	CSCC	93/90	Ischemic storke	PCR	PBMC/DNA	2.29(1.26 ~ 4.13)
Müller J 2003	Denmark	CSCC	193/368	Stroke	PCR	PBMC/DNA	1.35(0.74 ~ 2.46)
Sessa R 2003	Italy	CSCC	18/33	Ischemic stroke,TIA	PCR	PBMC/DNA	5.98(1.68 ~ 21.31)
Wohlschlaeger J 2005	German	CSCC	9/23	Ischemic stroke	PCR	Carotid plaque/DNA	2.75(0.15 ~ 49.36)
Sessa R 2003	Italy	CSCC	18/33	Ischemic stroke,TIA	PCR	Carotid plaque/DNA	1.84(0.56 ~ 6.05)
Katsenis C 2001	Greece	CSCC	20/15	Sympotomatic carotid disease	PCR	Carotid plaque/DNA	4.19(0.19 ~ 93.97)
LaBiche R 2001	USA	CSCC	37/57	Sympotomatic carotid disease	PCR	Carotid plaque/DNA	0.83(0.26 ~ 2.72)
Wohlschlaeger J 2005	German	CSCC	9/23	Ischemic stroke	IHC	Carotid plaque	0.14(0.01 ~ 1.27)
Lastas A 2004	Lithuania	CSCC	141/59	Sympotomatic carotid disease	IHC	Carotid plaque	0.88(0.33 ~ 2.38)
Neureiter D 2003	German	CSCC	20/40	Ischemic stroke	IHC	Carotid plaque	9.0(2.32 ~ 34.88)
Jahromi BS 2003	Canada	CSCC	57/13	Stoke,TIA	IHC	Carotid plaque	0.6(0.18 ~ 2.07)

CSCC, cross-sectional case control; PCR, polymerase chain reaction; TIA, transient ischemic attack; IHC, immunohistochemical method; PBMC, peripherial blood mononuclear cell.

### Determination of anti-C.pneumoniae IgG in serum

**Figure 2:**
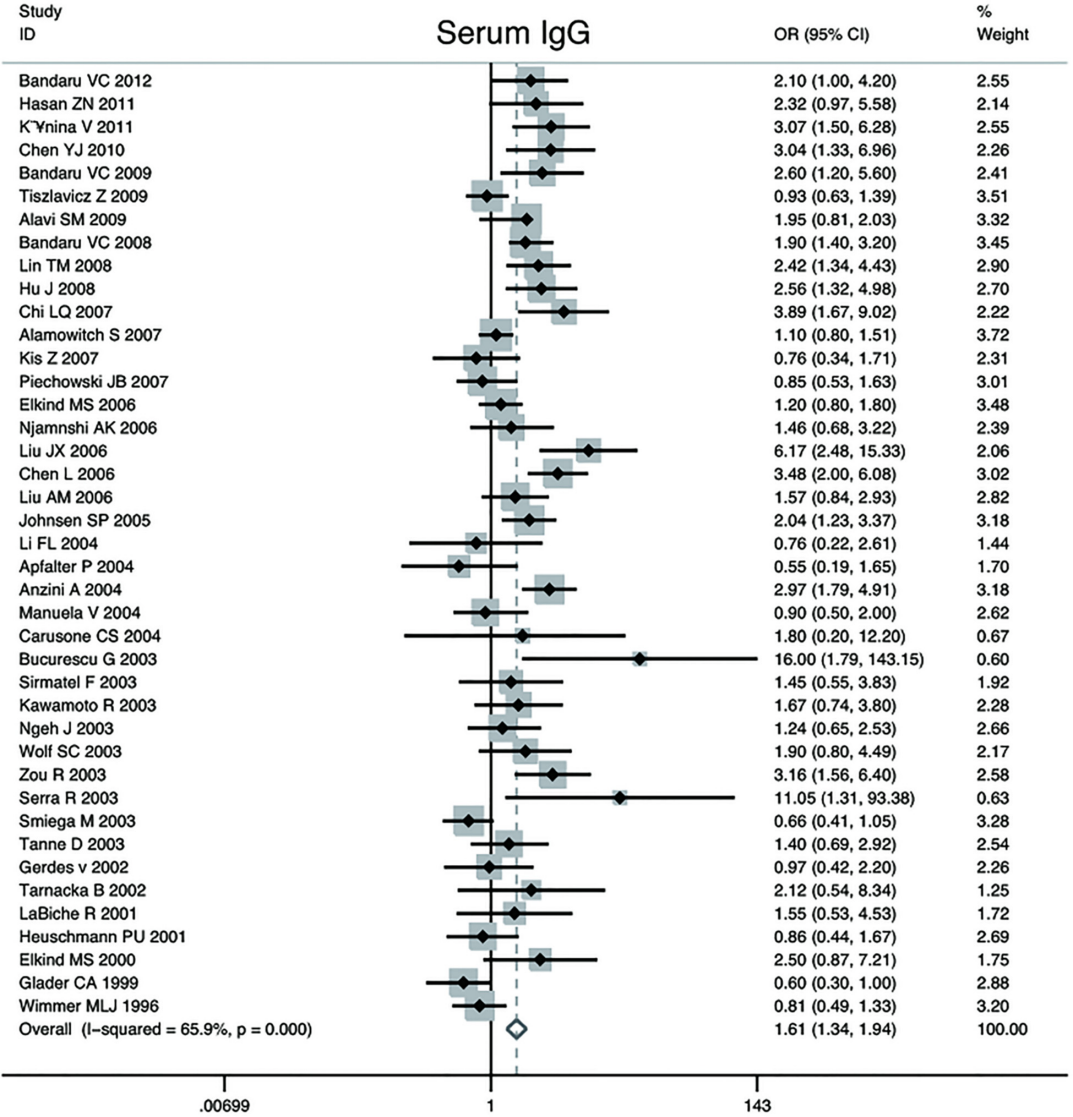
Forest plot on the random-effects of CV disease associated with C.pneumoniae infection by IgG seropositivity.

**Figure 3:**
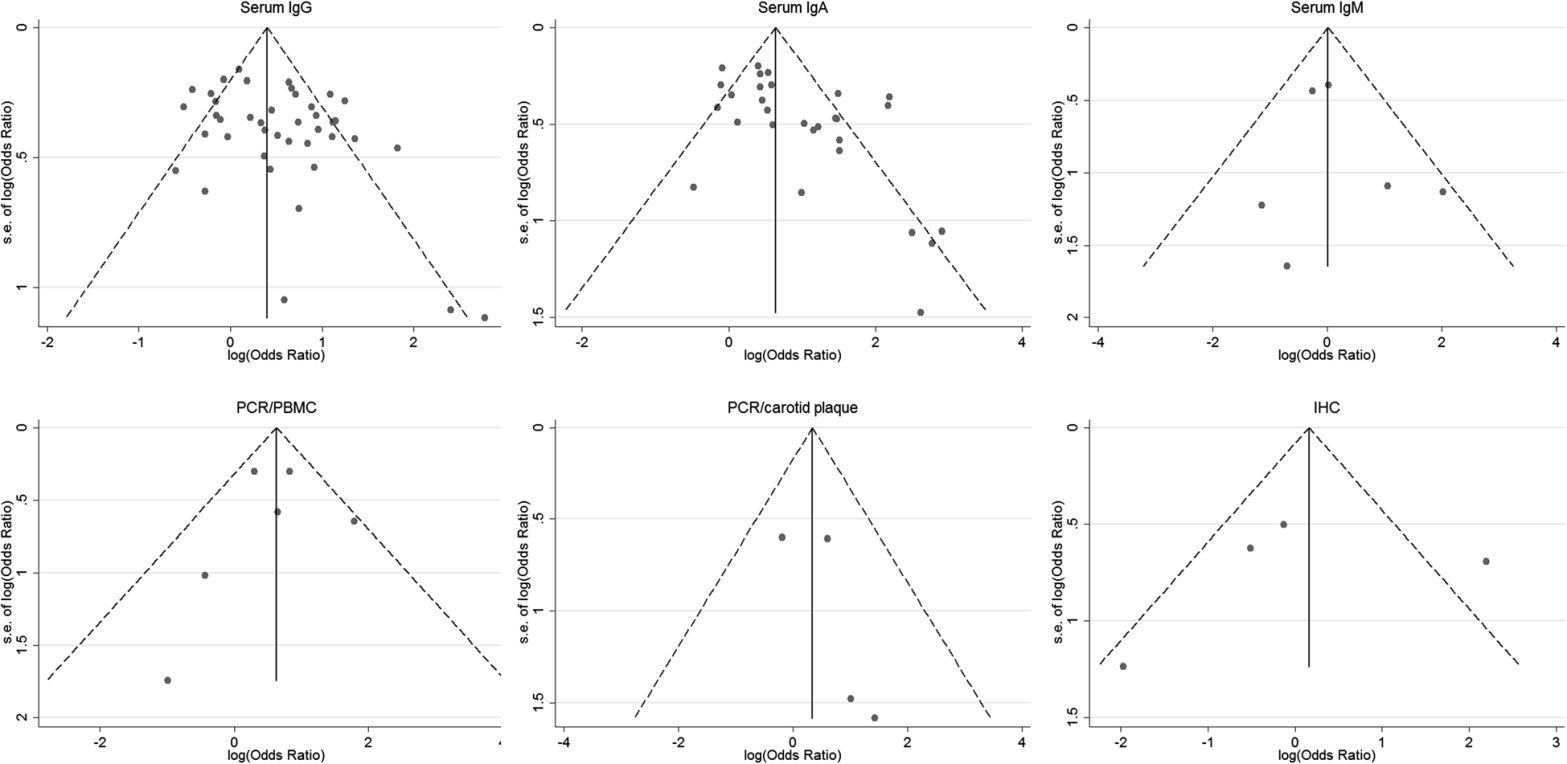
Funnel plots for publication bias detection.

Forty-two studies – thirty-six cross-sectional, five nested case control studies and one cohort study -- compared the association of CV disease group versus control groups with serum anti-C.pneumoniae IgG titers. The number of CV cases in the case–control studies ranged from 18 to 483, with a total of 4240; while the number of subjects in control groups ranged from 15 to 483, with a total of 7493. The overall estimated OR of case–control studies based on random effect was 1.61 (95% CI, 1.34 to 1.94; p = 0.00) and the cross-sectional studies demonstrated a more significant positive association with OR = 1.74 (95% CI, 1.43 to 2.10; p = 0.00). The Q statistic suggested an excessive heterogeneity between case–control studies (p, 0.00), with I^2^ = 65.9%. Furthermore, sensitivity analysis showed that the results were not affected by sequential exclusion of any particular study. No available methods favored the presence of publication bias. The Begger’s test yielded a P-value of 0.83 (zc = 1.58) and the Egger’s fixed effects regression suggested a marginally significant evidence for publication bias (t = 2.12; p = 0.04). Forest plot was shown in Figure 2. Funnel plot was shown in Figure 3. Considering the result yielded by Egger’s test, we had to conclude that the hypothesis of publication bias could not be further considered as valid. Applying“trim and fill” method to evaluate publication bias obtained a corrected OR = 1.62 (95% CI :1.34 to 1.95; p = 0.00), which suggested correction for potential publication bias did not materially alter the combined risk estimate. The one and only cohort study by Katie AC et al., with sample size of 1612 cases, which was not enrolled in this pooled analysis, reported an RR = 0.88 (95% CI: 0.52 to 1.47).

### Determination of anti-C.pneumoniae IgA in serum

**Figure 4:**
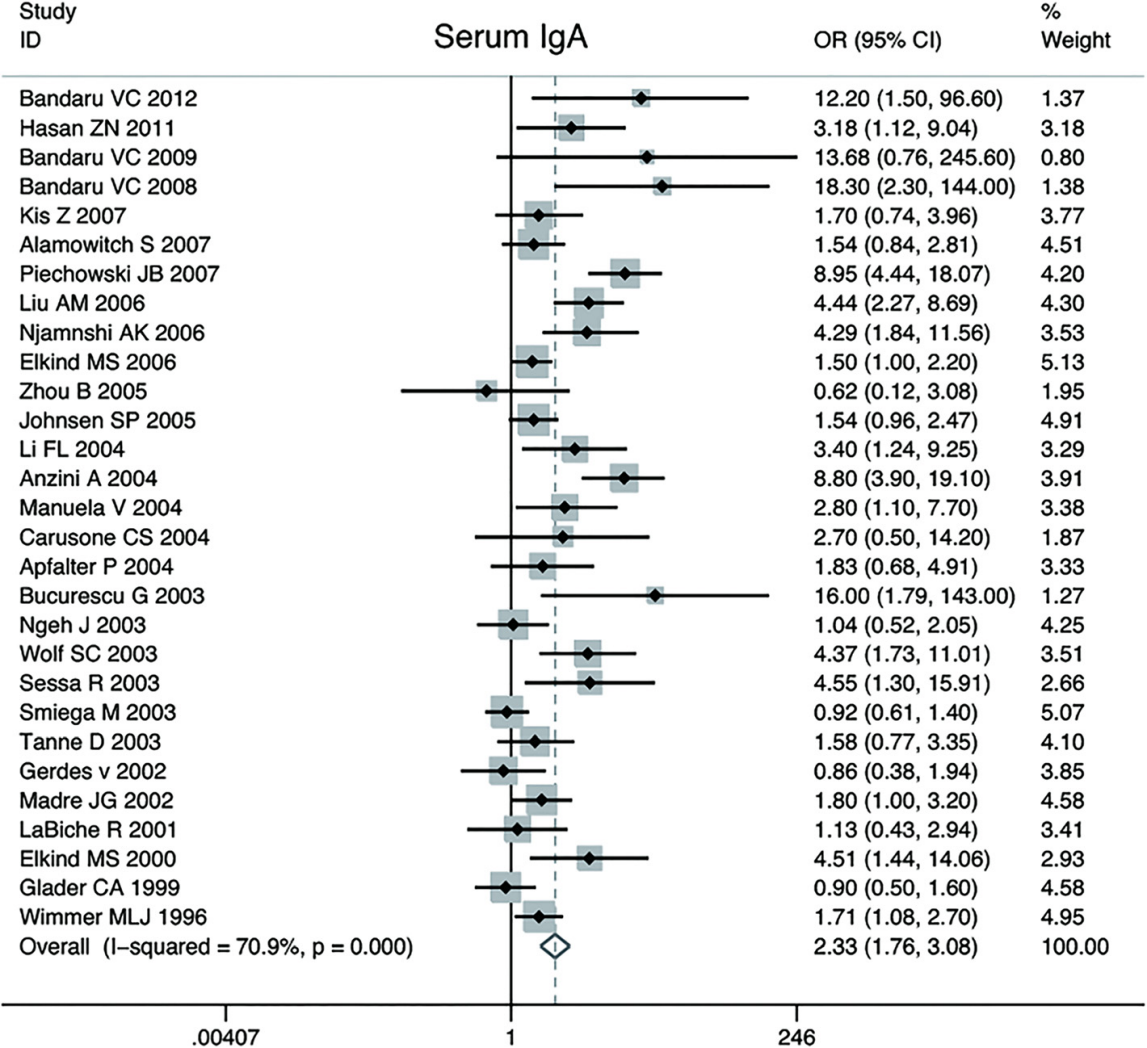
Forest plot on the random-effects of CV disease associated with C.pneumoniae infection by IgA seropositivity.

Thirty-one publications reported data on the association of C.pneumoniae infection and CV disease based on anti-C.pneumoniae IgA sero-positivity. The number of CV cases in the case–control studies ranged from 18 to 483, with a total of 2950; and the number subjects of control groups ranged from 25 to 483, with a total of 6759. With random effect model, C.pneumoniae infection was statistically associated with an increased risk of CV events in twenty-nine case–control studies (OR, 2.33; 95% CI: 1.76 to 3.08; p = 0.00), and the cross-sectional studies demonstrated a summary OR = 2.87 (95% CI: 2.11 to 3.91; p = 0.00). The heterogeneity between all case–control studies was substantial (p = 0.00; I^2^ = 70.9%). Furthermore, we observed the result was not affected by exclusion of any specific study from the pooled analysis. Forest plot was shown in Figure 4. Besides, Begger’s (zc = 2.55; p = 0.01) and Egger’s test (t = 3.15; p = 0.00) indicated presence of publication bias among those studies. The shape of the funnel plot did reveal some asymmetry (Figure 3). The adjusted pooled estimates by“trim and fill”method was OR 2.33 (95% CI: 1.76 ~ 3.08; p = 0.00), which suggested missing publications did not materially alter the combined risk estimate. The two prospective cohort studies were not included in the pooling analysis: one study (Elkind MS et al.), with a size of 1625, showed a positive relationship with RR = 1.30 while the other cohort study (Katie AC et al.) obtained RR = 0.67, suggesting a combined estimate effects on the association between C.pneunoniae infection with CV disease.

### Determination of serum anti-C.pneumoniae IgM

**Figure 5:**
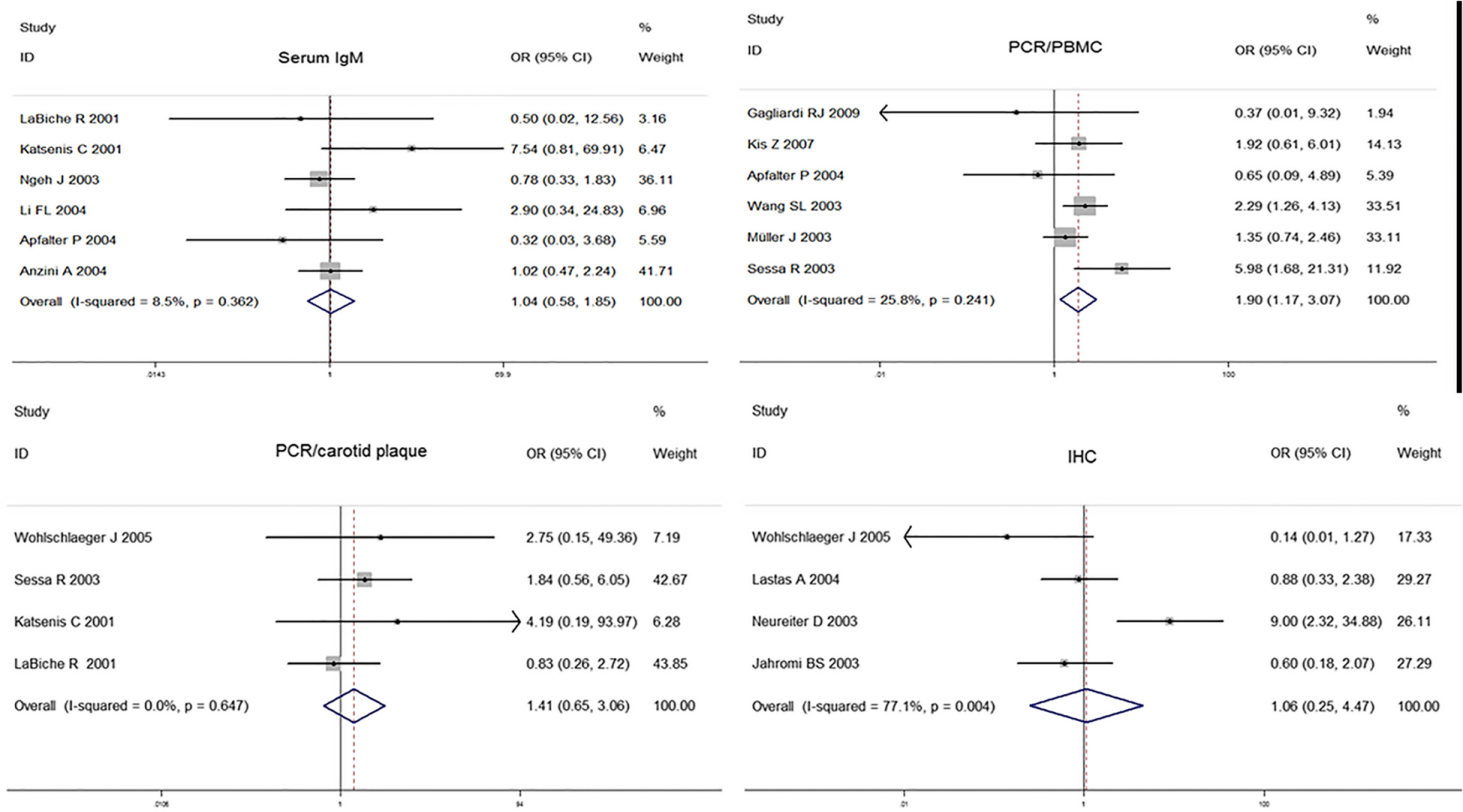
Forest plots on the random-effects of CV disease associated with C.pneumoniae infection on IgM seropositivity, PCR and IHC methods.

Serum anti-C.pneumoniae IgM titers were measured in six sero-epidemiologic case–control studies. The number of CV cases in the studies ranged from 20 to 141, with a total of 519; while the number of subjects of control groups ranged from 15 to 192, with a total of 454. The pooled estimate was 1.02 (95% CI: 0.47 to 2.24; p = 0.90). No significant heterogeneity was found among the six studies (p = 0.36; I^2^ = 8.5%).The Begger’s (zc = 0.00; p = 1.00) and Egger’s tests (t = 0.54; p = 0.62) suggested no publication bias, which were in accordance with the funnel plot. The forest plot was shown in Figure 5 and funnel plot was shown in Figure 3.

### PCR Detection of C.pneumoniae in PBMC

In six case–control studies, the peripheral blood cells were collected to detect C.pneumoniae DNA by PCR technique. The size of case groups ranged from 18 to 193, with a total of 460; while the size of control groups ranged from 30 to 368, with a total of 632. The overall OR for the association of C.pneumoniae with CV disease based on DNA detection was estimated to be 1.90 (95% CI: 1.17 to 3.07; p = 0.01). The homogeneity was observed among six studies (p = 0.24; I^2^ = 25.8%).The Begger’s (zc = 0.00; p = 1.00) and Egger’s tests (t = −0.35; p = 0.74) did not show any publication bias, and asymmetry did not occur in the funnel plot. The forest plot was shown in Figure 5. Funnel plot was shown in Figure 3.

### PCR detection of C.pneumoniae in carotid biopsies

There were four studies using PCR to detect the bacterium in carotid samples. The total number of samples in case groups was 84 and the number in control groups was 128. One by Katsenis et al., which obtained the highest OR had the lowest weighting, followed by study by Wohlschlaeger et al. Taken together, the studies reported an OR of 1.41 (95% CI: 0.65 to 3.06; p = 0.39), providing no evidence for a statistically significant association between CV disease and C.pneumoniae exposure. The studies were homogeneous among the studies (p = 0.64; I^2^ = 0.0%). The Begger’s and Egger’s test supported that the publication bias unlikely (zc = 1.02, p = 0.31; t = 1.34, p = 0.31). The forest plot was shown in Figure 5. Funnel plot was shown in Figure 3.

### Immunohistochemical studies

Four studies, included 227 total events of CV disease and 227 control cases, detected the C.pneumoniae antigen using IHC in carotid plaque. The study by Neureiter et al. obtained the highest OR. There was no evidence to support that C.pneumoniae in carotid plaque contributed the CV disease (OR, 1.06; 95% CI: 0.25 to 4.47; p = 0.93). The Q test yields a significant P-value of 0.00, suggesting a significant heterogeneity with I^2^ = 77.1%. Moreover, sensitivity analysis showed that the exclusion of any study did not alter the pooled result. Neither the Begger’s (zc = 0.34; p = 0.73) nor the Egger’s test (t = −0.31; p = 0.79) showed any publication bias. The forest plot was shown in Figure 5. Funnel plot was shown in Figure 3.

### Subgroup analysis

There have been some serological studies reporting the results by etiological subtypes of stroke(TOAST criteria: large artery atherosclerosis, cardioembolism, small artery occlusion, other or undefined etiology). In order to exclude the influence of different study methods, the research carried out subanalysis of association between C.pneumoniae infection and CV disease on seropositivity of IgG and IgA , respectively.

Seven studies on seropositivity of anti-C.pneumoniae IgG were included. Subanalysis showed a positive statistically significant result for the CV cases with large artery disease versus the control (OR, 1.91; 95% CI: 0.92 to 3.97; p = 0.08) ,which was much more significant than ischemic stroke of other etiologies: for cardioembolism(OR, 1.87; 95% CI: 0.59 to 5.97; p = 0.29), for small artery occlusion (OR = 1.01; 95% CI: 0.63 ~ 1.64; p = 0.96); for other etiology (OR, 0.80; 95% CI: 0.23 to 2.72; p = 0.72) and for undefined etiology (OR, 1.52; 95% CI: 0.69 ~ 3.33; p = 0.30).

There were five research reports dedicating the relationships between seropositivity of anti-C.pneumoniae IgA and stroke by TOAST criteria. Subanalysis of these studies suggested the advanced OR in large artery atherosclerosis versus control was 2.25 (95% CI: 1.12 to 4.51; p = 0.02), which was much more significant than cardioembolism groups (OR, 1.58; 95% CI: 0.92 to 2.72; p = 0.10), small artery occlusion (OR, 0.77; 95% CI: 0.21 to 2.92; p = 0.71), other etiology (OR, 1.07; 95% CI: 0.43 to 2.68; p = 0.88) or the undefined etiology (OR, 1.89; 95% CI: 0.61 to 5.88; p = 0.27).

In one study (Neureiter D etc.) using IHC to detect C.pneumoniae infection, all the cases suffered from ischemic stroke of large artery-atherosclerosis according to the TOAST criteria, and it showed a significant association with OR = 9.0. The studies detecting C.pneumoniae infection based on serum IgM or by PCR did not investigate the association by stroke subtypes.

### Meta-regression on study-level covariates

In an attempt to explain the large amount of heterogeneity in the sero-epidemiological results, meta-regression was applied, where we attempted to use as explanatory variables, various study-level covariates. We finally considered two potential covariates: proportion of males in cases and the mean age of cases. Unfortunately, multivariate regression demonstrated no significant between the two moderators and only bits of ORs heterogeneity originates from the different age and male percents in each study.

## Discussion

The notion that C.pneumoniae infection may be responsible for atherosclerosis and vascular accidents is not new. Previous meta-analysis just addressed the association between C.pneumoniae and coronary artery disease (CAD) or atherosclerosis based on sero-epidemiologic studies [60]. Even though CV disease shares many risk factors with CAD because of the underlying atherosclerotic mechanism, it was much more heterogeneous for the comprehensive manifestation and multifactorial etiology. Large amount of studies have identified an apparent linkage between C.pneumoniae infection and CV accidents, as several studies failed to demonstrate consistent associations. The present study was promoted for the absence of any published meta-analysis on the association between C.pneumoniae infection and CV disease. In consideration of the diversity of laboratorial methods and interpretative criteria, we enrolled all available studies to date using different markers of chronic C.pneumoniae infection rather than sero-epidemiological studies only to evaluate the relationship point to point.

### Antibody investigation

Serum antibody detection is the most commonly used assay to diagnose acute or chronic C.pneumoniae infection, including the markers: IgG, IgA and IgM. Each type of immunoglobin has the specific kinetic feature. In primary infection patients, IgM antibody appears about 2 to 3 weeks after the onset of infection and is generally undetectable after 2 to 6 month in serum. IgG antibody does not reach high title until 6 weeks after the onset of illness, with a half-life of weeks to months. In case of reinfection, IgM antibody may not appear and the level of IgG titer increase within a week [61]. Thus, the isolated elevated IgG can only be interpreted as a previous antigenic contact and past infection, with the exception of IgG elevation between two samples or an especially high level in one sample represents a current infection, whereas IgM may not be detectable in specimens in early course of illness or reinfection. IgA has a biological half-life less than 7 days and the presence has been associated with current or chronic infection. Although debated, high IgA titres has been suggested to be a more valid and reliable measure of chronic active infection compared with IgG [18].

Studies enrolled in this meta-analysis used ELISA or MIF to detect IgG, IgA or IgM, with the titer cutoff points of 1/16 or higher by MIF. In the meta-analysis of case–control studies, the elevated titer of IgA or IgG showed a significant relationship with CV disease, while the IgM did not. These findings are consistent with reports that most people have antigenic contact with C.penumoniae early in their lives and that re-infections are the norm, accounting for the absence of IgM in reinfection [62]. There were only two prospective cohort studies published to date evaluating the relation between C.pneumoniae infection and CV disease, while one used ELISA and the other one used MIF as detecting method, obtaining contradictory results by IgA and a negative result by IgG. In general, prospective cohort studies are considered superior to case–control and cross-sectional studies, since prospective studies investigate temporal relations and can indicate whether the exposure variable preceded the outcome variable. In contrast, in cross-sectional studies, the potentially causal exposure variable and the outcome variable are determined at the same or close moment, which makes it difficult to distinguish between them, since pneumonia is the most common post-stroke infection, which mostly occur in acute phase of stroke [63]. Nevertheless, if participants in prospective studies are infected in the period between blood collection and stroke manifestation, the exposure status will also be misclassified, which leads to excessively underestimation of the relation. After all, despite the statistical significance of the positive association between C.pneumoniae and CV disease, its causal relevance to CV disease remains uncertain for inadequate literature amount of prospective cohort studies.

### PCR studies in PBMC

Recently, C.pneumoniae DNA detection by PCR technique has been shown to be a valid diagnostic method and apparently more reliable than other methods. C.pneumoniae may localize in atheromatous cardiovascular tissues, peripheral blood monocytes and atheromatous lesions of arteries [52,56]. These organisms and positive monocytes may contribute to total antibody production. PCR test allows the active current infection diagnosis, being negative for the patients previously contaminated and already healed. The presence of circulating DNA of C.pneumoniae may represent a persistent systemic infection, but this method must be performed with great caution to prevent false-positive and false-negative results. Our evaluation of studies with PCR of PBMC showed that C.pneumoniae DNA confers an odds ratio for CV disease of approximately 1.90 (95% CI:1.17 to 3.07) and the results of the five studies were homologous. The significant results from sero-epidemiological studies and PCR detection in PMBC all contribute to the hypothesis that a common secondary C.pneumoniae infection eventually leading to atheroma formation and plaque rupture, which is in accordance with previous studies [64].

### Tissue diagnosis by PCR and IHC

C.pneumoniae can directly infected cells of vessel wall, leading to latent residence infection. That is why the DNA and antibody of the pathogen has been identified in atheromatous tissues by use of various methods [4]. The pooled analysis of four studies with PCR on carotid plaque biopsy showed a combined OR, suggesting no significant difference in the prevalence of C.pneumoniae infection between cases and controls. IHC offers the advantage of preserving tissue morphology and permitting localization of the infectious agent to specific areas and tissues. In the four studies using IHC as detecting method, only one showed a strong relationship with a high OR = 9.0. Despite this, no significant relationship was found by pooled analysis (OR = 1.06). The discrepancy between the results from peripheral blood and atheromatous plaques may be attributed to several factors. Wohlschlaeger J etc. evaluated different cerebral aortic biopsy in patients with CV disease and observed the highest C.peneumoniae prevalence using PCR in right cerebral vascular artery (20%), and lower in carotid artery (11.1%) or basilar artery (10%). What’s more, by IHC, they observed the highest prevalence in the basilar artery (30%) and right middle cerebral artery (30%), but lowest in the carotid artery (11.1%) [56]. Thus, it is questionable if bare signals observed from carotid samples reflect systematically the authentic condition of the patients. Another explanation is the fact that both patients with CV disease and control subjects was atherosclerosis, the only difference was whether they were symptomatic. Part of the control subjects have undergone carotid endarterectomy are liable to have advanced atherosclerosis and at the highest risk of current CV events. After all, the lack of a true gold standard of PCR or IHC technique to date makes it difficult to assess these methods. A recent study, in which C.pneumoniae seroconversion assay and PCR methods were evaluated for diagnosis of C.pneumoniae infection during an outbreak in a military community, suggested that the sensitivity of PCR was lower than that of serologic antibody testing [65].

### Association by subgroups of strokes

Stroke is a disease of different etiologies (i.e., large artery disease, cardioembolism or small vessel disease). Investigations in etiological subtypes of ischemic stroke may help to clarify the possible role of C.pneumoniae in CV disease, which has only been addressed in studies of small sample size. Previous studies indicated that seropositivity to C.pneumoniae is strongest in patients with stroke of large artery atherosclerosis, which contributes to 10 ~ 20% of strokes, as defined in Acute Stroke Treatment (TOAST) criteria, but less frequent in patients with a stroke due to small-vessel occlusion [66]. In our meta-analysis, this issue was confirmed by seropositivity of IgG and IgA, which is consistent with the hypothesis that C.pneumoniae contributes to atherosclerosis. One of in-situ studies using IHC technique researched the relation by large artery atherosclerosis, and strengthened the relation more [57]. Furthermore, the results also suggested C.pneumoniae might be effective in cardioembolism by elevated serum IgG (OR = 1.87) as well as IgA (OR = 1.58), which deserve more investigation in future studies. Because stroke of cardioembolism results from coronary atherosclerosis in many cases, it is plausible that these patients would also be associated with C.pneumoniae infection. The issue whether C.pneumoniae infection promotes stroke of small vessel disease or other etiology remains questionable for inadequate sample size.

The development of atherosclerotic CV disease involves a series of steps: initiation, progression, plaque formation, plaque rupture or erosions, and thrombosis. Compelling data indicate that C.pneumoniae infection does contribute to the latter stage of carotid atherosclerosis instead of the earlier stage, since it might promote atherogenesis and eventually trigger acute cardiovascular events [64,67]. Some studies also suggested patients infected within a week before the onset of stroke were more likely to develop cerebral infarcts [68]. The pathogen may gain access to the vasculature during local infection and exacerbate atherosclerosis either directly or indirectly. Direct effects include cell lysis, lipid oxidization and proliferation of smooth muscle cells. Indirect systematic effects may involve induction of acute phase proteins expression, establishment of a prothrombotic state and promotion arteriosclerosis by activating monocytes [69,70]. Furthermore, C.pneumoniae was suggested to trigger specific cell-mediated immunity within plaques and promote plaques rupture by enhancing the production of matrix-degrading metalloproteinase, which was considered to be responsible for fibrous cap rupture [71]. Activated immune cells also produced cytokines and cytotoxic antibodies, which in turn, lead to the local perpetuation of the vessel wall damage. Further researchers proposed the bacterium act in cooperation with conventional risk factors and genetic predisposition, which insufficient for disease generation alone [56]. Therefore, whether a specific pathogen will initiate or accelerate the complications of atherosclerosis maybe determined by the complex interactions of those complex factors. It would thus appear that further research will be needed before we can predict how C.pneumoniae influence atherogenesis and its potential interaction with other pathogenic factors.

The major strength of our meta-analysis is that we enrolled all available cross-sectional and prospective studies using various detection methods, which enhanced comprehensiveness of the findings and reduced the likelihood of selection bias. However, several limitations should be acknowledged. First, at present, the assessment of chronic active C.pneumoniae infection is difficult and there is no wholly satisfactory method for diagnosis. In this meta-analysis, we defined the diagnosis standard in various approach according to the recommendations from the Centers for Disease Control and Prevention and the Laboratory Centre for Disease Control [61]. Second, heterogeneity in sero-epidemiological studies is substantial. The variability in serum sample dilutions and in the method used by commercial laboratories may partially account for this heterogeneity. It is also recognized that cross-reactivity between C.pneumoniae and other micro-organism may occur in serology. Other possible explanations include small sample sizes, degree of the results adjustment, time of blood collection and stage of the CV disease. In addition, prospective studies typically can provide more information on the clinical relevance. Regrettably, only two prospective cohort studies investigated CV disease were eligible and yielded contradictory results, thus insufficient to demonstrate a causal relationship on this issue.

## Conclusions

In the present meta-analysis, seropositivity for anti-C.pneumoniae IgG, IgA and C.pneumoniae DNA were found significantly associated with increased CV disease in case–control studies, and the serum IgM was not. Meanwhile, data from the present study did not show a significant correlation between CV disease and in-situ detection of C.pneumoniae in arteries specimens. Thus, the finding or not of an association depends on the method employed. In addition, sub-analysis of available studies showed C.pneumoniae is strongest associated with patients with strokes of large artery atherosclerosis, but less frequent in patients with a stroke due to cardioembolism and no clear association were found for other types of ischemic stroke. In conclusion, assessment of different microbiological technique methods and more prospective cohort studies about various stroke subtypes are needed in future to confirm the causal relationship between C.pneumoniae infection and CV disease.

## Competing interests

The authors declare that they have no competing interests.

## Authors’ contributions

CJ: Design Initiation, Methodology, Data collection, Analysis, Manuscript writing. ZMJ: Design, Methodology, Data collection, Quality assessment, Supervision, Manuscript revision. ZZN: Analysis, Manuscript revision. MGT: Methodology, Supervision. SZW: Manuscript revision. All authors read and approved the final manuscript.

## Pre-publication history

The pre-publication history for this paper can be accessed here:

http://www.biomedcentral.com/1471-2377/13/183/prepub
